# Predictive Factors for Failure of Noninvasive Ventilation in Adult Intensive Care Unit: A Retrospective Clinical Study

**DOI:** 10.1155/2020/1324348

**Published:** 2020-08-01

**Authors:** Qimin Chen, Ming Liu, Bo Liu, Wei Li, Daixiu Gao, Lulu Xie, Yu Wu, Liang Li, Ying Liu, Ying Wang, Tang Yan, Yuanyi Liu, Yumei Cheng, Xu Liu, Feng Shen

**Affiliations:** Department of Critical Care Medicine, The Affiliated Hospital of Guizhou Medical University, Guiyang, China

## Abstract

**Background:**

Noninvasive ventilation (NIV) has been reported to be beneficial for patients with acute respiratory failure in intensive care unit (ICU); however, factors that influence the clinical outcome of NIV were unclarified. We aim to determine the factors that predict the failure of NIV in critically ill patients with acute respiratory failure (ARF). *Setting*. Adult mixed ICU in a medical university affiliated hospital. *Patients and Methods*. A retrospective clinical study using data from critical adult patients with initial NIV admitted to ICU in the period August 2016 to November 2017. Failure of NIV was regarded as patients needing invasive ventilation. Logistic regression was employed to determine the risk factor(s) for NIV, and a predictive model for NIV outcome was set up using risk factors.

**Results:**

Of 101 included patients, 50 were unsuccessful. Although more than 20 variables were associated with NIV failure, multivariate logistic regression demonstrated that only ideal body weight (IBW) (OR 1.110 (95%1.027–1.201), *P*=0.009), the maximal heart rate during NIV period (HR-MAX) (OR 1.024 (1.004–1.046), *P*=0.021), the minimal respiratory rate during NIV period (RR-MIN) (OR 1.198(1.051–1.365), *P*=0.007), and the highest body temperature during NIV period (T-MAX) (OR 1.838(1.038–3.252), *P*=0.037) were independent risk factors for NIV failure. We set up a predictive model based on these independent risk factors, whose area under the receiver operating characteristic curve (AUROC) was 0.783 (95% CI: 0.676–0.899, *P* < 0.001), and the sensitivity and specificity of model were 68.75% and 71.43%, respectively, with the optimal cut-off value of 0.4863.

**Conclusion:**

IBW, HR-MAX, RR-MIN, and T-MAX were associated with NIV failure in patients with ARF. A predictive model based on the risk factors could help to discriminate patients who are vulnerable to NIV failure.

## 1. Introduction

Acute respiratory failure (ARF) is one of the most common reasons for intensive care unit (ICU) admission [1, 2]. Mechanical ventilation is the most effective therapy for those patients [1]. During the past two decades, invasive mechanical ventilation (IMV) has been the major supportive method for ARF in ICU [2]. But endotracheal intubation or tracheotomy is needed for IMV, which will inevitably increase the patient's pain or cause some harm to the patient. IMV also increases the risk of lower respiratory infection such as ventilator associated pneumonia (VAP) [3]. These disadvantages of IMV are worrying to more and more intensivists when IMV is being used nowadays. Contrarily, noninvasive mechanical ventilation (NIV), in which artificial airway is not necessarily established, is much more comfortable and less harmful for the patient, and more convenient than with IMV. Application of NIV in patients with AFR was seen as an important development in the field of mechanical ventilation for the past twenty years [4]. Data from a population-based study which involved more than 10 million cases of ARF from 2000 to 2009 showed that proportions of patients with and without chronic obstructive pulmonary disease (COPD) receiving NIV increased from 3.5% to 12.3% and 1.2% to 6%, respectively [5]. Whenever NIV is used, however, caution must be taken into account that NIV failure may occur in some patients, which was reportedly associated with adverse outcomes of patients [6].

NIV failure is often defined as the need for invasive mechanical ventilation with endotracheal intubation [7–9]. In spite of convenience and ease, failure is not uncommon with NIV. Clinical literature reported that the incidence of NIV failure varies greatly from 5% to 60%, depending on causes of ARF and the morbidity, etc. [10, 11]. Incidence of NIV failure was reported approaching about 50% in patients with community-acquired pneumonia and acute respiratory distress syndrome (ARDS) [8, 9]. Results of clinical trial demonstrated that NIV failure was independently associated with some bad clinical outcomes such as increased morbidity and mortality [8–12]. Therefore, it is crucial to identify the factors being able to predict patients who cannot benefit from NIV as early as possible, so that patients could be timely endotracheally intubated and ventilated with IMV in cases it will be necessary [8]. Although a lot of clinical trials described/studied application of NIV in patients with COPD [13–16], fewer paid close attention to the use of NIV in ICU patients with ARF caused by diseases besides COPD.

In this retrospective clinical study, we aimed to calculate the incidence of NIV failure in critically ill patients and tried to find out these factors that could predict NIV failure, so that we could discriminate the patient as early as possible who may benefit from IMV rather than NIV.

## 2. Methods

### 2.1. Study Population

This retrospective clinical trial was conducted in a 46-bed mixed ICU of a medical university affiliated hospital between August 2016 and November 2017. Data were collected from those patients who initially received NIV because of ARF after ICU admission. The patients who were treated with IMV first and received NIV later were excluded.

### 2.2. Inclusion Criteria

Patients who were included must meet the criteria as follows:

1. More than 18 years of age
2. Having indications for NIV : ARF resulting from all kinds of diseases such as pneumonia, ARDS, COPD, asthma, and cardiogenic pulmonary edema

### 2.3. Exclusion Criteria

Patients who were less than 18 years old, who were pregnant, who had facial deformity and facial surgery, who had upper gastrointestinal hemorrhage and severe vomiting, who were unconscious with inability to protect the airway/to clear respiratory secretions, who had high risk of aspiration, who were severely hemodynamically instable, and who had severe hypoxia (with oxygenation index less than 100 mmHg) were excluded from the study.

### 2.4. NIV Protocol

Patients were treated with NIV after ICU admission if they had the indication (mentioned above). A proper full-face mask (PHILIPS vision AF531, USA), secured with head straps, was used to connect the respirator (PHILIPS Respironics V60, Respironics California, Inc, USA) [17] to the patient. Bi-level positive airway pressure (BIPAP) was the most used model in our ICU. Parameters including respiratory rate (RR), pressure support level (PS), and fraction of inspired oxygen (FiO_2_) were adjusted at the discretion of the attending clinician and the respiratory therapist according to the patient's situation, maintaining the total RR less than 25 breaths per minute (BPM) and peripheral oxygen saturation S_P_O_2_ more than 90%. A certain expiratory positive airway pressure (EPAP) was used during NIV procedure. Patients were generally not given sedation. If they appeared agitative and felt uncomfortable with the mask, then low dose of dexmedetomidine was intravenously applied with caution [6]. All patients were monitored with SpO_2_, electrocardiography, state of consciousness, etc. With improvement of the patient's situation, NIV model was correspondingly changed to continuous positive airway pressure (CPAP), and gradual decrease of PS level could be considered and, if effective, then NIV weaning could be carried out.

### 2.5. Data Collection

Once patients were admitted to ICU, they were monitored with electrocardiography and S_P_O_2_ and with vital signs including invasive blood pressure, heart rate (HR), respiratory rate, and body temperature (axillary temperature). Some important data were recorded for each patient, including age, gender, actual body weight, height, main diagnoses, comorbidities, cause of ARF, and APACHEII score. Arterial blood gas analysis, peripheral blood cell count, hemoglobin value, liver and renal function, and electrolyte were all measured at ICU admission. Daily input and output volume and daily medications and usage of vasoactive drugs for every patient were also recorded every day at 08 : 00 am during NIV period or the first six days.

From the first day to the sixth day of NIV, minimal and maximal values of parameters mentioned above were calculated for each patient.

### 2.6. Endpoint and Definition

#### 2.6.1. Criteria for NIV Success and Failure

  Success criteria: improvement of tachypnea, a decrease of ≥20% in respiratory rate, an improvement in arterial blood gases with pH value of more than 7.35, a decrease of PaCO_2_ of ≥15% compared with spontaneous breathing, maintaining SaO_2_% ≥90% (with and without oxygen), and improvement of the patient' dyspnea and comfort [18].
  Failure criteria [19]: requirement of intubation after NIV intervention based on the following criteria: respiratory or cardiac arrest, failure to maintain a P_a_O_2_/F_i_O_2_ of >100 mmHg, development of conditions necessitating intubation to protect the airway (coma or seizure disorders) or to manage copious tracheal secretions, inability to correct dyspnea, lack of improvement of signs of respiratory muscle fatigue, and hemodynamic instability without response to fluids and vasoactive agents.

## 3. Statistical Analysis

### 3.1. Analysis of Predictive Factors for NIV Outcome

Statistical analysis was performed using SPSS 18.0. Counting variables such as sex and comorbidity were statistically described with frequency and percentage. Normality of the continuous variables was tested by Kolmogorov-Smirnov test. Continuous variables with normal distribution were described as mean and standard deviation (SD), and continuous variables with skewed distribution were described as median（Quartile）. Difference in normally distributed continuous variables between groups was compared using group *t*-test or corrected *t*-test, according to results of Levene's homogeneity test of variance. In skewed distributed variables, difference between groups was analyzed using the Mann–Whitney test. Variables that were significantly different between groups were further applied for multivariate logistic regression, in which the stepwise regression method was used to screen factors that independently influenced the outcome of NIV. *P* < 0.05 was considered to be statistically significant.

### 3.2. Methods for Construction and Evaluation of the Predictive Model for NIV Outcome

After determining the independent predictors through the multivariate logistic regression, we randomly chose 70% of all data used as training data and the other 30% of data were regarded as testing dataset, among which the training dataset was used for building prediction model through a logistic regression and the testing dataset was employed to evaluate the prediction accuracy of the model [20]. ROC analysis was used to determine the optimal cut-off point for the prediction model through the maximum method for Youden index, and the sensitivity, specificity, and Youden index were calculated, so as to evaluate the predictive efficiency of the optimal cut-off point.

## 4. Results

### 4.1. Description for NIV Success

During our clinical study period, a total of 101 patients underwent NIV, of whom 51 patients were successful and another 50 were unsuccessful. The success incidence was 50.50% and the failure incidence was 49.5%.

### 4.2. Etiologies of the Patients

Pneumonia, severe acute pancreatitis, COPD, and malignant tumor were the most common etiologies of our patients, and trauma, septic shock, postoperative status, and heart disease were also commonly seen in two groups (Table 1).

### 4.3. Univariate Regression Analysis for NIV Failure

In unsuccessful patients, they had relatively higher height (163.92 vs. 159.2, *P*=0.003) and higher ideal body weight (IBW) (*P*=0.004). Patients who experienced NIV failure appeared to have higher heart rate at the first day of ICU admission and had higher maximal heart rate during NIV treatment(*P*=0.004, 0.003, respectively). Maximal and minimal respiratory rate appeared in unsuccessful patients (*P*=0.003, 0.034, respectively), and these patients had maximal body temperature during NIV period (*P*=0.014). During the therapy period, unsuccessful patients had much more total input volume (3333.50 (2578.75, 4497.25) ml vs. 2762.00 (2267.00, 3652.00) ml) and intravenous input volume (2571.00 (1689.25, 3899.00) ml vs. 1890.00 (1117.00, 2750.00) ml) than successful ones (*P*=0.025, 0.03, respectively). More patients in failure group experienced positive infusion balance (38 (58.46%) vs. 27 (41.54%), *P*=0.016) and more patients needed norepinephrine (14 (82.35%) vs. 3(17.65%), *P*=0.003) than patients in successful group (Supplemental Data file, Table S1).

Some other parameters, such as ALT, AST, and total bilirubin, were all different between the two groups (Supplemental Data file, Table S1).

### 4.4. Multivariate Logistic Regression Analysis for NIV Failure

Variables which were significantly different between failure patients and success patients were employed to carry out multivariate regression analysis, so as to screen out the independent risk factors for NIV failure. Results showed that ideal body weight, maximal heart, minimal respiratory rate, and maximal body temperature during NIV treatment period were independent risk factors for NIV failure in critically ill patients with ARF (Table 2).

### 4.5. Building Up the Predictive Model for NIV Outcome

Data from all patients were randomly divided into model training dataset (*n* = 71) and testing dataset (*n* = 30). Then, a predictive model for NIV outcome based on the independent risk factors in the multivariate analysis was developed and calculated as follows:

(1) $$\begin{array}{c} \text{Logit}\left(P\right)\;=\;\frac{1}{\left[1\;+\text{ exp}\left(32.263\;-\;0.130\;\times \;\mathrm{IBW}\;-\;0.018\;\times \;\mathrm{HR}-\text{MAX }-\;0.175\;\times \;\mathrm{RR}-\text{MIN }-\;0.509\;\times \;T-\text{MAX}\right)\right]}, \end{array}$$

where IBW is ideal body weight; HR-MAX is the maximal heart rate during NIV treatment; RR-MIN is the minimal respiratory rate during NIV period; and T-MAX is the highest body temperature during NIV treatment.

#### 4.5.1. Optimal Cut-Off Value Decision in the Predictive Model for NIV Outcome

**Figure 1:**
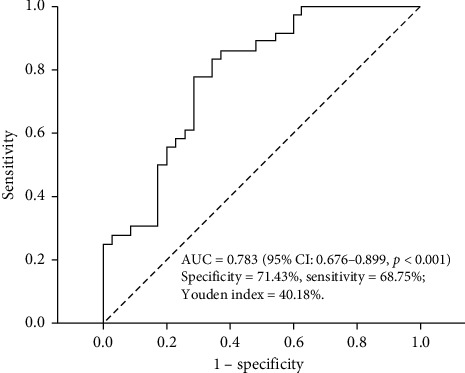
ROC of predictive model for NIV outcome.

Using receiver operating characteristic curve (ROC), we know that the overall model performance was satisfactory, with an AUROC of 0.783 (95% CI: 0.676–0.899, *P* < 0.001). The maximal value of Youden index was 49%, and the corresponding optimal cut-off value of Logit(*P*) was 0.4863 (Figure 1).

#### 4.5.2. Efficacy Evaluation of the Predictive Model for NIV Outcome

The predictive efficacy of optimal cut-off value of model was evaluated by sensitivity, specificity, and Youden index. The sensitivity and specificity of model were 68.75% and 71.43%, respectively. Youden Index was 40.18% (Table 3).

## 5. Discussion

Among 101 patients, 50 of them failed NIV, with failure incidence of 49.5%, which was higher than the 37.3% (44/118) in patients with acute cardiogenic pulmonary edema [21], the 17.1% (13/76) in patients with obesity [22], and the 38.4% (43/112) in patients with ARF after cardiac surgery, respectively [23]. This discrepancy of failure incidence may be associated with different etiologies and other factors [24]. In this retrospective study, we found that many variables were different between success and failure patients, but there were only ideal body weight, fastest heart rate, slowest respiratory rate, and highest body temperature during NIV treatment period that were independent risk factors for NIV failure in critically ill patients with ARF. With an optimal cut-off value of Logit(*P*) of 0.4863, the predictive model for NIV failure had a sensitivity of 68.75% and a specificity of 71.43%, respectively, indicating that the predictive efficacy was satisfactory. Since the late 1980s, NIV has increasingly been used in ARF resulting from a variety of reasons, such as after cardiac surgery [23], influenza infection [25], chest trauma [26], COPD [15], after abdomen surgery [27], and after solid organ transplantation [28]. As NIV failure was associated with higher risk of mortality and of other bad outcomes in patients with ARF [6], and even associated with a higher possibility of death compared to patients who initially received invasive mechanical ventilation [25], it is very important to recognize the profiles of those patients who very possibly failed NIV. Although univariate analysis results demonstrated that many parameters were associated with NIV failure, multivariate analysis showed higher IBW, maximal heart rate, minimal respiratory rate, and higher body temperature were independent risk factors for NIV failure.

Higher IBW is a strong predictor for NIV failure in our study, meaning obesity is a risk factor associated with NIV failure. Obesity often causes reduced lung and chest wall compliance, increasing airway resistance, and overweight subjects could be at increased risk of alveolar collapse [18], all of which could make obese patient more vulnerable to NIV failure [29]. But in a retrospective study led by Liu et al., BMI ≥25 kg/m^2^ was a predictor of NIV success in patients with postextubation acute respiratory failure after cardiac surgery [23], of which results seemed to contradict ours. We think the possible reason might be a different disease being included in this other study. Our patients might have more complicated comorbidity than patients have in another study. Maximal heart rate during NIV treatment is another independent risk factor for NIV failure, which was consistent with previous studies [30, 31]. We think that, during NIV period, persisted tachycardia often meant other problems such as fever, hemodynamic instability, electrolyte disturbance, etc., coexisting which resulted in dramatic increase in oxygen consumption. Interestingly, results of our study revealed that patient's minimal respiratory rate during NIV was also independently associated with NIV failure. In some previous clinical trials, there were no significant differences in regard to respiratory rate between NIV success and NIV failure [23, 32]. But in some other investigations, a higher respiratory rate of more than 35 breaths/min at administration in COPD patients[11], or an increase of respiratory rate 1 hour after NIV, was reported as a risk factor for NIV failure in postoperative ARF patients and hematological malignancies in ARF and in ALI patients [33–35]. In our analysis, a higher and a lower respiratory rate during NIV period were both associated with NIV failure demonstrated by univariate regression analysis (*P* = 0.034, 0.003, respectively). In multivariate regression analysis, however, only minimal respiratory rate during NIV treatment was a high risk factor for NIV failure (OR 1.198 (95% CI 1.051–1.365), *P* = 0.007). We suppose that a minimal respiratory rate (very low respiratory rate) often means obvious fatigue of respiratory muscle [36] and makes patients more vulnerable to NIV failure.

Finally, our data showed that highest body temperature during NIV period was an independent risk factor for NIV failure. So far, very fewer studies explored the relationship between body temperature and NIV outcome. It was tested that pneumonia and other kinds of infections were independently associated with NIV failure [18, 23, 37]. So we speculate that, during NIV, a gradually elevated body temperature often means an infection, aggravating the patients' respiratory work. By using these four independent risk factors and the patients' dataset, we set up a predictive model for NIV outcome in ARF patients. With an AUROC of 0.783, Youden index of 49%, and optimal cut-off value of 0.4863, the predictive model performance was satisfactory, with sensitivity and specificity of 68.75% and of 71.43%, respectively. The model is expected to help us discriminate those critically patients early who might fail NIV, avoiding some NIV failure-associated undesirable complications.

There are some limitations in our analysis. First, the study was conducted at our single ICU, making selection bias inevitable. Second, NIV management, NIV failure judgement, and the indication and timing for endotracheal intubation were all mainly based on the decision of the attending physician, which possibly created bias in therapeutic strategy of individualized subject.

In conclusion, our retrospective analysis found that IBW, maximal heart rate, minimal respiratory rate, and maximal body temperature during NIV treatment were independent risk factors for NIV failure in critically ill patients. A predictive model for NIV outcome with satisfactory sensitivity and specificity offered us a tool to discriminate patients early who were at risk for NIV failure and avoid some complications associated with NIV failure.

## Figures and Tables

**Table 1:** Etiologies of patients who were admitted to our ICU and received NIV.

Etiologies	Total (*n* = 101)	Success group (*n* = 51)	Failure group (*n* = 50)
Pneumonia, n	19	9	10
Severe acute pancreatitis, *n*	15	10	5
COPD, *n*	14	9	5
Malignant tumour, *n*	10	6	4
Trauma, *n*	7	5	2
Septic shock, *n*	6	1	5
Postoperative status *n*	3	1	2
Heart disease, *n*	5	3	2
liver cirrhosis, *n*	2	1	1
Urinary system infection, *n*	5	3	2
Other pulmonary diseases, *n*	3	1	2
CPR, *n*	1	1	0
SLE, *n*	1	0	1
Adiposis, *n*	1	0	1
Sicca syndrome, *n*	1	0	1
Central nerve system diseases, *n*	2	0	2
Uremia, *n*	1	0	1
Others, *n*	5	1	4

NIV, noninvasive ventilation; COPD, chronic obstructive pulmonary disease; CPR, cardiac pulmonary resuscitation; SLE, systemic lupus erythematosus.

**Table 2:** Multivariate analysis of the risk factors for noninvasive ventilation failure.

Variables	OR and 95%CI	*P* value
IBW (kg)	1.110(1.027, 1.201)	0.009
HR-MAX (bpm)	1.024(1.004, 1.046)	0.021
RR-MIN (bpm)	1.198(1.051, 1.365)	0.007
T-MAX (°C)	1.838(1.038, 3.252)	0.037

Data were presented as median (95% of confidence interval, CI); IBW, ideal body wight; HR-MAX, the maximal heart rate during NIV period (beats per minute, bpm); RR-MIN, the minimal respiratory rate during NIV period (breath per minute, bpm); T-MAX, the highest body temperature during NIV period.

**Table 3:** Evaluation of predictive model for NIV outcome.

Dataset	Predictive results	NIV outcome		Total	Sensitivity (%)	Specificity (%)	Youden index (%)
		Success	Failure				
Training dataset	Success	25	8	33	71.43	77.78	49.21
	Failure	10	28	38			
	Total	35	36	71			

Testing dataset	Success	11	4	15	68.75	71.43	40.18
	Failure	5	10	15			
	Total	16	14	30			

## Data Availability

The data used to support the findings of this study are available from the corresponding author upon request.

## Abbreviations

ICU: Intensive care unit
NIV: Noninvasive ventilation
ROC: Receiver operating characteristic curve
IBW: Ideal body weight
HR-MAX: Maximal heart rate during NIV period
RR-MIN: Minimal respiratory rate during NIV period
T-MAX: Highest body temperature during NIV treatment
ARF: Acute respiratory failure.

## Supplementary Materials

Supplementary Materials Table S1: univariate analysis of the risk factors for noninvasive ventilation failure.
