# Supplementary material for: Predictive Factors for Failure of Noninvasive Ventilation in Adult Intensive Care Unit: A Retrospective Clinical Study
Source: Can Respir J. 2020 Aug 1;2020:1324348. doi: 10.1155/2020/1324348 (PMC7421696; doi:10.1155/2020/1324348)
Supplement: Supplementary Materials — Table S1: univariate analysis of the risk factors for noninvasive ventilation failure. [file 1324348.f1.pdf]

Table S1 Univariate analysis of the risk factors for non-invasive ventilation failure

| Variable | Total （n=101) | NIV outcome | | *$\chi^2$/t/Z value* | *p* value |
|---|---|---|---|---|---|
| | | NIV failure (n=50) | success (n=51) | | |
| Gender,n(%) | | | | | |
| Male | 57(56.44) | 33(57.89) | 24(42.11) | 3.684 [a] | 0.055 |
| Female | 44(43.56) | 17(38.64) | 27(61.36) | | |
| Age(years) | 61.91±18.43 | 62.78±18.64 | 61.06±18.36 | 0.467 [b] | 0.641 |
| Wight(kg) | 57.38±10.94 | 58.52±11.31 | 56.27±10.55 | 1.034 [b] | 0.304 |
| Height(cm) | 161.53±8.14 | 163.92±7.76 | 159.20±7.89 | 3.033 [b] | 0.003 |
| BMI($kg/m^2$) | 21.92±3.58 | 21.67±3.44 | 22.17±3.73 | 0.709 [b] | 0.480 |
| IBW(kg) | 56.44±6.15 | 58.21±5.92 | 54.71±5.92 | 2.972 [b] | 0.004 |
| APACHⅡ score at ICU admission | 21.06±6.25 | 21.70±5.90 | 20.43±6.57 | 1.020 [b] | 0.310 |
| Interval between admission of hospital and ICU(d) | 1.00(0.00,6.50) | 1.00(0.00,6.00) | 1.00(0.00,9.00) | 0.257 [c] | 0.797 |
| Duration of NIV (d) | 4.00(2.00,6.00) | 4.00(2.00,6.00) | 4.00(3.00,6.00) | 0.038 [c] | 0.970 |
| Morbidity, n (%) | | | | | |

| | | | | | | |
|---|---|---|---|---|---|---|
| Hypertension | | | | | | |
| Without | 65(64.36) | 32(49.23) | 33(50.77) | 0.005 | a | 0.941 |
| With | 36(35.64) | 18(50.00) | 18(50.00) | | | |
| CHD | | | | | | |
| Without | 92(91.09) | 44(47.83) | 48(52.17) | 1.164 | a | 0.281 |
| With | 9(8.91) | 6(66.67) | 3(33.33) | | | |
| COPD | | | | | | |
| Without | 90(89.11) | 46(51.11) | 44(48.89) | 0.853 | a | 0.356 |
| With | 11(10.89) | 4(36.36) | 7(63.64) | | | |
| Immunosuppressive agents | | | | | | |
| Without | 100(99.01) | 49(49.00) | 51(51.00) | 1.030 | a | 0.310 |
| With | 1(0.99) | 1(100.00) | 0(0.00) | | | |
| HR-D1(bpm) | 110.99±23.96 | 115.82±22.19 | 106.25±24.90 | 2.037 | b | 0.044 |
| HR-MAX (bpm) | 117.50±23.36 | 124.26±20.66 | 110.86±24.13 | 2.995 | b | 0.003 |
| MAP-D1(mmHg) | 96.28±18.10 | 96.61±18.55 | 95.95±17.83 | 0.184 | b | 0.854 |
| MAP-MIN (mmHg) | 81.90±15.47 | 80.49±18.64 | 83.29±11.59 | 0.911 | b | 0.365 |

| | | | | | | |
|---|---|---|---|---|---|---|
| MAP-MAX(mmHg) | 103.60±15.89 | 103.79±16.20 | 103.41±15.74 | 0.118 | b | 0.906 |
| RR-D1(bpm) | 26.00(21.50,33.00) | 26.00(22.00,33.25) | 25.00(21.00,31.00) | 1.419 | c | 0.156 |
| RR-MIN(bpm) | 20.00(18.50,23.00) | 21.00(19.00,25.00) | 20.00(17.00,21.00) | 2.993 | c | 0.003 |
| RR-MAX(bpm) | 29.00(25.00,34.50) | 30.00(26.00,38.00) | 27.00(23.00,33.00) | 2.122 | c | 0.034 |
| T-D1(℃) | 37.22±0.81 | 37.32±0.97 | 37.12±0.61 | 1.242 | b | 0.217 |
| T-MIN(℃) | 36.72±0.51 | 36.74±0.62 | 36.71±0.38 | 0.236 | b | 0.814 |
| T-MAX(℃) | 37.72±0.90 | 37.94±1.07 | 37.50±0.63 | 2.494 | b | 0.014 |
| $SPO_2$-D1(%) | 96.00(86.50,99.00) | 95.00(85.50,98.00) | 97.00(90.00,100.00) | 1.398 | c | 0.162 |
| $SPO_2$-MIN(%) | 93.00(86.00,96.00) | 91.00(84.00,93.25) | 94.00(90.00,98.00) | 2.787 | c | 0.005 |
| SPO2_MAX | 100.00(96.00,100.00) | 98.50(96.00,100.00) | 100.00(98.00,100.00) | 2.405 | c | 0.016 |
| PH-D1 | 7.42(7.33,7.48) | 7.42(7.30,7.48) | 7.42(7.37,7.47) | 0.479 | c | 0.632 |
| PH_MIN | 7.38(7.29,7.45) | 7.35(7.27,7.45) | 7.40(7.33,7.45) | 1.319 | c | 0.187 |
| PH-MAX | 7.48(7.43,7.53) | 7.49(7.40,7.53) | 7.48(7.44,7.52) | 0.418 | c | 0.676 |
| $PaCO_2$-D1 | 33.00(26.50,46.00) | 32.50(24.00,43.50) | 35.00(30.00,47.00) | 1.356 | c | 0.175 |
| $PaCO_2$-MIN | 31.00(25.50,38.00) | 29.50(24.00,37.25) | 32.00(27.00,39.00) | 1.414 | c | 0.157 |
| $PaCO_2$-MAX | 40.00(34.00,57.00) | 37.00(32.00,58.25) | 41.00(36.00,52.00) | 1.444 | c | 0.149 |

| | | | | | | |
|---|---|---|---|---|---|---|
| $HCO_3^-$-D1 | 23.55±9.24 | 22.52±9.60 | 24.55±8.86 | 1.104 | b | 0.272 |
| HCO3_MIN | 22.37±8.93 | 21.30±9.10 | 23.43±8.72 | 1.203 | b | 0.232 |
| $HCO_3^-$-MAX | 29.74±10.64 | 27.90±10.84 | 31.54±10.22 | 1.738 | b | 0.085 |
| $FiO_2$_D1 | 50.00(40.00,80.00) | 50.00(40.00,80.00) | 45.00(40.00,80.00) | 0.628 | c | 0.530 |
| $FiO_2$-MAX | 55.00(40.00,95.00) | 60.00(43.75,100.00) | 50.00(40.00,80.00) | 1.562 | c | 0.118 |
| Total input volume（ml）-D1 | 1497.00(874.50,2776.00) | 1686.00(895.00,3290.50) | 1330.00(665.00,2610.00) | | | |
| Total input volume-MAX(ml） | 3080.00(2375.00,4124.50) | 3333.50(2578.75,4497.25) | 2762.00(2267.00,3652.00) | 2.245 | c | 0.025 |
| Cumulative input volume during NIV(ml） | 8213.00(4306.00,11955.00) | 9238.00(5666.25,12426.00) | 6995.00(4002.00,11318.00) | 1.345 | c | 0.179 |
| Intravenous intput volume-D1(ml) | 1225.00(682.50,2468.50) | 1242.00(745.00,2906.25) | 1100.00(652.00,2048.00) | 1.015 | c | 0.310 |
| Intravenous input volume-MAX(ml) | 2161.00(1437.50,3640.50) | 2571.00(1689.25,3899.00) | 1890.00(1117.00,2750.00) | 2.174 | c | 0.030 |
| Cumulative intravenous volume during NIV(ml) | 5420.00(2970.00,9013.50) | 6664.50(3433.25,9402.00) | 3890.00(2739.00,8008.00) | 1.854 | c | 0.064 |
| Total output volume-D1（ml） | 1280.00(660.00,2262.50) | 1350.00(775.00,1925.00) | 1200.00(600.00,2570.00) | 0.391 | c | 0.696 |
| Total output volume-MIN(ml) | 1100.00(500.00,1700.00) | 1025.00(452.50,1470.00) | 1200.00(600.00,1900.00) | 1.063 | c | 0.288 |
| Total output volume-MAX(ml) | 2815.00(2025.00,3970.00) | 2775.00(2087.50,3902.50) | 2850.00(1900.00,4180.00) | 0.425 | c | 0.671 |

| | | | | | | |
|---|---|---|---|---|---|---|
| Cumulative output volume（ml） | 6760.00(3965.00,11115.00) | 6300.00(3742.50,10765.00) | 6850.00(4000.00,13250.00) | 0.696 | c | 0.486 |
| Urinary volume-D1(ml) | 900.00(485.00,1425.00) | 1000.00(452.50,1412.50) | 850.00(500.00,1700.00) | 0.224 | c | 0.823 |
| Urinary volume（ml）-MIN | 700.00(300.00,1100.00) | 610.00(242.50,1100.00) | 730.00(400.00,1200.00) | 1.040 | c | 0.298 |
| Urinary volume-MAX（ml） | 2000.00(1315.00,2800.00) | 2000.00(1075.00,2657.50) | 2000.00(1400.00,3150.00) | 1.077 | c | 0.282 |
| ALT-D1(U/L) | 28.00(16.99,61.08) | 37.32(17.43,94.45) | 22.39(16.31,51.34) | 1.583 | c | 0.114 |
| ALT-MAX | 31.52(20.68,111.33) | 50.94(20.71,195.08) | 27.60(19.39,57.20) | 2.000 | c | 0.045 |
| AST-D1(U/L) | 44.92(31.12,106.45) | 56.96(37.20,161.97) | 40.80(25.69,68.00) | 2.364 | c | 0.018 |
| AST-MAX(U/L) | 54.94(35.74,181.28) | 65.00(42.43,274.61) | 44.90(30.41,81.10) | 2.731 | c | 0.006 |
| Cr-D1(μmmol/L) | 82.81(58.59,177.24) | 85.35(63.69,173.47) | 82.81(53.71,181.10) | 0.734 | c | 0.463 |
| Cr-MAX (μmol/L) | 91.84(62.29,194.43) | 101.48(68.33,233.57) | 83.00(58.01,194.00) | 1.209 | c | 0.227 |
| TB-D1(mmol/L) | 15.85(10.28,35.94) | 18.60(11.40,52.55) | 14.70(8.51,22.40) | 2.092 | c | 0.036 |
| TB-MAX (mmol/L) | 20.35(11.84,43.83) | 24.85(14.85,64.84) | 17.40(10.50,28.58) | 2.326 | c | 0.020 |
| ALB-D1(g/L) | 32.20±6.09 | 30.84±6.19 | 33.52±5.75 | 2.255 | b | 0.026 |
| ALB-MIN(g/L) | 29.32±5.11 | 27.96±5.06 | 30.65±4.84 | 2.727 | b | 0.008 |
| PA-D1(mg/L) | 126.74±69.78 | 117.71±74.55 | 135.59±64.27 | 1.292 | b | 0.200 |
| PA-MIN(mg/L) | 102.24±64.36 | 89.71±67.27 | 114.52±59.48 | 1.964 | b | 0.052 |

| | | | | | | |
|---|---|---|---|---|---|---|
| TRO-D1(ng/ml) | 0.03(0.02,0.10) | 0.02(0.01,0.10) | 0.04(0.02,0.10) | 1.423 | c | 0.155 |
| TOR-MAX (ng/ml) | 0.04(0.02,0.15) | 0.04(0.01,0.14) | 0.05(0.03,0.16) | 1.396 | c | 0.163 |
| BNP-D1(pg/ml) | 2422.00(518.23,9800.00) | 1922.00(461.00,10649.00) | 2717.00(665.45,10253.50) | 0.635 | c | 0.525 |
| BNP_MAX(pg/ml) | 3426.00(870.25,13910.75) | 3084.50(1071.50,15969.00) | 3932.50(777.23,10253.50) | 0.228 | c | 0.819 |
| Na-D1(mmol/L) | 139.50±6.20 | 139.65±5.78 | 139.36±6.65 | 0.230 | b | 0.819 |
| Na-MIN(mmol/L) | 137.64±4.73 | 137.96±4.66 | 137.32±4.82 | 0.682 | b | 0.497 |
| Na-MAX(mmol/L) | 142.49±5.99 | 143.28±6.27 | 141.71±5.66 | 1.322 | b | 0.189 |
| K-D1(mmol/L) | 4.17±0.86 | 4.13±0.96 | 4.21±0.76 | 0.496 | b | 0.621 |
| K-MIN(mmol/L) | 3.86±0.75 | 3.90±0.88 | 3.81±0.60 | 0.580 | b | 0.564 |
| K-MAX(mmol/L) | 4.61±0.83 | 4.66±0.95 | 4.56±0.69 | 0.635 | b | 0.527 |
| LAC-D1(mmol/L) | 2.10(1.00,3.55) | 2.50(1.05,4.30) | 2.00(0.93,2.88) | 1.704 | c | 0.088 |
| LAC-MAX(mmol/L) | 2.50(1.50,3.55) | 2.60(1.80,4.30) | 2.15(1.23,2.98) | 2.068 | c | 0.039 |
| WBC（×10$^9$/L）-D1 | 11.07(7.76,15.35) | 11.91(8.39,16.17) | 9.42(7.72,13.57) | 1.644 | c | 0.100 |
| WBC（×109/L）-MIN | 8.77(6.27,12.46) | 9.69(5.97,14.25) | 8.13(6.47,11.50) | 1.372 | c | 0.170 |
| WBC（×10$^9$/L）-MAX | 12.85(8.97,18.79) | 14.53(10.16,24.96) | 10.82(8.50,15.44) | 2.656 | c | 0.008 |
| HB-D1（g/L） | 114.09±32.94 | 113.02±34.83 | 115.14±31.30 | 0.322 | b | 0.748 |

| | | | | | | |
|---|---|---|---|---|---|---|
| HB-MIN（g/L） | 103.15±31.99 | 103.44±34.02 | 102.86±30.20 | 0.090 | [b] | 0.929 |
| OI-D1 (mmHg) | 170.00(116.78,261.25) | 144.75(91.88,215.38) | 188.00(137.50,288.75) | 2.619 | [c] | 0.009 |
| OI-MIN (mmHg) | 148.89(97.50,175.00) | 119.06(78.75,163.13) | 162.22(128.00,210.00) | 3.424 | [c] | <0.001 |
| Number of patients not using NE-D1, n(%) | 92(91.09) | 43(46.74) | 49(53.26) | | [a] | 0.075 |
| Number of patients using NE-D1, n(%) | 9(8.91) | 7(77.78) | 2(22.22) | 3.159 | | |
| Number of patients not using NE during NIV, n(%) | 84(83.17) | 36(42.86) | 48(57.14) | 8.823 | [a] | 0.003 |
| Number of patients using NE during NIV, n(%) | 17(16.83) | 14(82.35) | 3(17.65) | | | |
| Number of patients not using dopamine-D1, n(%) | 100(99.01) | 50(50.00) | 50(50.00) | 0.990 | [a] | 0.320 |
| Number of patients using dopamine-D1, n(%) | 1(0.99) | 0(0.00) | 1(100.00) | | | |
| Number of patients not using dopamine during NIV, n(%) | 100(99.01) | 50(50.00) | 50(50.00) | 0.990 | [a] | 0.320 |
| Number of patients using dopamine during NIV, n(%) | 1(0.99) | 0(0.00) | 1(100.00) | | | |

| | | | | | |
|---|---|---|---|---|---|
| Number of patients with different liquid balance | | | | | |
| Infusion balance-D1, n(%) | | | | | |
| Negative | 36(35.64) | 17(47.22) | 19(52.78) | 0.117[a] | 0.733 |
| Positive | 65(64.36) | 33(50.77) | 32(49.23) | | |
| Infusion balance-during NIV, n(%) | | | | | |
| Negative | 36(35.64) | 12(33.33) | 24(66.67) | 5.852[a] | 0.016 |
| Positive | 65(64.36) | 38(58.46) | 27(41.54) | | |

Data were presented as mean ± SD, or median (interquartile range), or number (percentage); Statistical analysis were completed by a Pearson Chi square test (a), Group T test (b), or by Mann-Whitney U test.

BMI body mass index; IBW ideal body wight; CHD,coronary heart disease; COPD,chronic obstructiv pulmonary disease; HR, heart rate; bpm,beat per minute; D1, the value at the first day of ICU admission; MAX, the maximal value during NIV treatmen; MIN, the minimal value during NIV treatment; RR, respiratory rate (breath per minute, bpm), T, temperature; ALT, alanine aminotransferase; AST, aspartate aminotransferase; Cr, serum creatinine; TB, total bilirubin; ALB, albumin; PA, prealbumin; TRO, troponin； BNP, brain natriuretic peptide； Na, sodium ion; K, potassium ion; LAC, serum lactic acid; WBC, peripheral white blood cell count; HB, hemoglobin; OI, oxygenation index; NE, norepinephrine. NIV, non-invasive mechanical ventilation.
